# 2-Benzyl-6-chloro-1-(4-methyl­phen­yl)-1*H*-indole-3-carbonitrile

**DOI:** 10.1107/S1600536811048422

**Published:** 2011-11-23

**Authors:** Qiao Yan, Xiuxiang Qi

**Affiliations:** a School of Pharmaceutical Science and Technology, Tianjin University, Tianjin 300072, People’s Republic of China

## Abstract

In the title compound, C_23_H_17_ClN_2_, the dihedral angle between the indole ring and the attached tolyl ring is 86.97 (8)°. Weak C—H⋯N(nitrile) hydrogen bonding, and C—H⋯π(aromatic) and short Cl⋯π(aromatic) [3.628 (1) Å] inter­actions consolidate the crystal packing.

## Related literature

For the synthesis of the title compound, see: Du *et al.* (2006 ▶). For its precursor, see: Jin *et al.* (2009 ▶). For related structures, see: Yang *et al.* (2011 ▶); Yan & Qi (2011*a* ▶,*b* ▶,*c* ▶). For standard bond lengths, see: Allen *et al.* (1987 ▶).
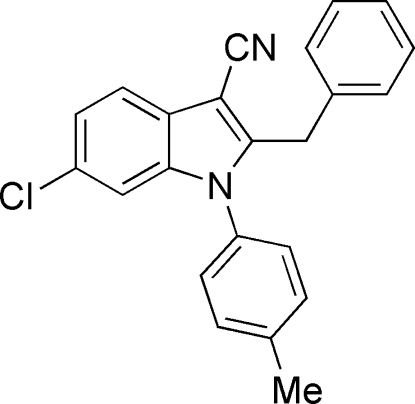


## Experimental

### 

#### Crystal data

C_23_H_17_ClN_2_
*M* *_r_* = 356.84
Monoclinic, 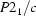

*a* = 10.0003 (10) Å
*b* = 9.8565 (8) Å
*c* = 18.539 (2) Å
β = 93.926 (9)°
*V* = 1823.1 (3) Å^3^
*Z* = 4
Mo *K*α radiation
μ = 0.22 mm^−1^
*T* = 113 K
0.20 × 0.16 × 0.10 mm

#### Data collection

Rigaku Saturn724 CCD diffractometer
Absorption correction: multi-scan (*CrystalClear-SM Expert*; Rigaku, 2009 ▶) *T*_min_ = 0.958, *T*_max_ = 0.979
18792 measured reflections
4382 independent reflections
1947 reflections with *I* > 2σ(*I*)
*R*_int_ = 0.079

#### Refinement

*R*[*F* ^2^ > 2σ(*F* ^2^)] = 0.041
*wR*(*F* ^2^) = 0.103
*S* = 0.85
4382 reflections
237 parameters
H-atom parameters constrained
Δρ_max_ = 0.35 e Å^−3^
Δρ_min_ = −0.30 e Å^−3^

### 

Data collection: *CrystalClear-SM Expert* (Rigaku, 2009 ▶); cell refinement: *CrystalClear-SM Expert*; data reduction: *CrystalClear-SM Expert*; program(s) used to solve structure: *SHELXS97* (Sheldrick, 2008 ▶); program(s) used to refine structure: *SHELXL97* (Sheldrick, 2008 ▶); molecular graphics: *SHELXTL* (Sheldrick, 2008 ▶); software used to prepare material for publication: *SHELXL97*.

## Supplementary Material

Crystal structure: contains datablock(s) global, I. DOI: 10.1107/S1600536811048422/zl2420sup1.cif

Structure factors: contains datablock(s) I. DOI: 10.1107/S1600536811048422/zl2420Isup2.hkl

Supplementary material file. DOI: 10.1107/S1600536811048422/zl2420Isup3.cml

Additional supplementary materials:  crystallographic information; 3D view; checkCIF report

## Figures and Tables

**Table 1:** Hydrogen-bond geometry (Å, °) *Cg* is the centroid of the C17–C22 ring.

*D*—H⋯*A*	*D*—H	H⋯*A*	*D*⋯*A*	*D*—H⋯*A*
C9—H9*A*⋯N2^i^	0.99	2.64	3.557 (3)	154
C14—H14⋯*Cg*^ii^	0.95	2.84	3.735 (2)	157

Symmetry codes: (i) 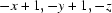; (ii) 
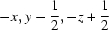
.

**Fig. 1.**
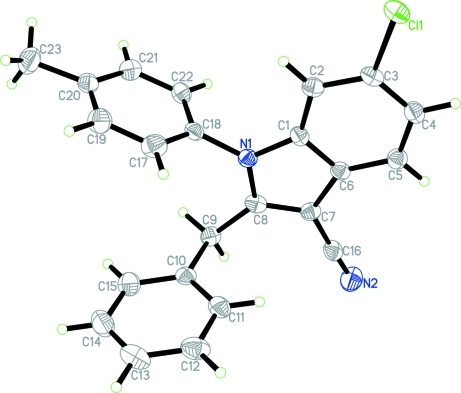
View of the title compound showing displacement ellipsoids at the 50% probability level.

**Fig. 2.**
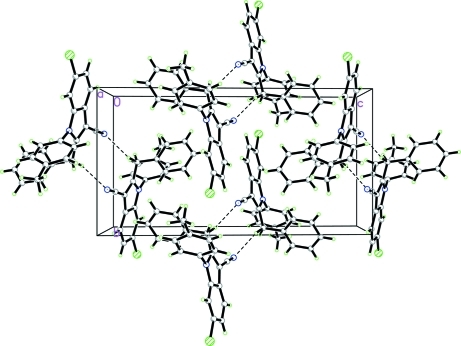
Molecular packing of the title compound, viewed down the a axis. The C—H···N H-bonding is represented by dashed lines.

**Fig. 3.**
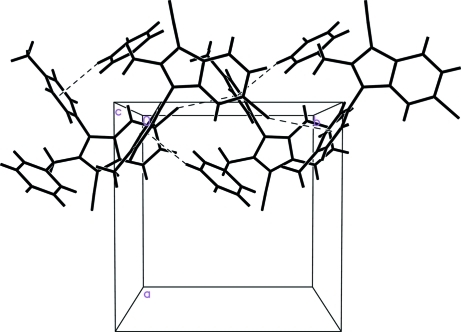
Molecular packing of the title compound, viewed down the c axis. The C—H···π and Cl···π interactions are represented by dashed lines.

## supplementary crystallographic information

### Comment

Indoles are widely applied in many fields such as pharmaceuticals. In order to disclose their structure-activity relationship, we have studied the crystal structures of a series of indole derivatives (Yan & Qi, 2011*a*,b,c). In our continuous investigation of indole derivatives, we herein report the structure of the title compound (Fig. 1). The indole ring forms an angle of 86.97 (8)° with the tolyl ring connected to the N1 atom. This is similar as in ethyl 2-benzyl-1-propyl-1*H*-indole-3-carboxylate [80.91 (5)°] reported by Yang *et al.* (2011), but greater than in 1-(4-methoxyphenyl)-2-methyl-1*H*-indole-3-carbonitrile [58.41 (4)°] (Yan & Qi, 2011*a*) and 1-(4-bromophenyl)-2-methyl-1*H*-indole-3-carbonitrile [58.85 (11)°] (Yan & Qi, 2011*b*) reported by our group. All bond distances and angles of the title compound are within the normal ranges (Allen *et al.*, 1987).

In the crystal packing, a weak intermolecular hydrogen-bond interaction occurs, affording a dimer with the nitrile N atom being a hydrogen-bond acceptor and the methylene being a hydrogen-bond donor (Table 1 & Fig. 2). Significant C—H···π interaction exists between H14 and the aromatic C17—C22 ring (*Cg*) (Table 1). A short Cl···π interaction is detected between Cl1 and the other face of the same toyly ring. These C—H···π and Cl···π interactions link molecules into columns along the *b* axis (Fig. 3).

### Experimental

The title compound was prepared according to the method of the literature (Du *et al.*, 2006). Colourless prisms of the title compound were grown by evaporation from a mixture of ethyl actate and petroleum ether.

### Refinement

All H atoms were positioned geometrically (C—H = 0.95, 0.98 and 0.99 Å) and refined as riding with *U*_iso_(H) = 1.2*U*_eq_(CH & CH_2_) or 1.5*U*_eq_(CH_3_).

### Crystal data

**Table:**

C_23_H_17_ClN_2_	*F*(000) = 744
*M**_r_* = 356.84	*D*_x_ = 1.300 Mg m^−^^3^
Monoclinic, *P*2_1_/*c*	Mo *K*α radiation, λ = 0.71073 Å
Hall symbol: -P 2ybc	Cell parameters from 6077 reflections
*a* = 10.0003 (10) Å	θ = 2.0–28.0°
*b* = 9.8565 (8) Å	µ = 0.22 mm^−^^1^
*c* = 18.539 (2) Å	*T* = 113 K
β = 93.926 (9)°	Prism, colorless
*V* = 1823.1 (3) Å^3^	0.20 × 0.16 × 0.10 mm
*Z* = 4	

### Data collection

**Table:**

Rigaku Saturn724 CCD diffractometer	4382 independent reflections
Radiation source: rotating anode	1947 reflections with *I* > 2σ(*I*)
multilayer	*R*_int_ = 0.079
Detector resolution: 14.22 pixels mm^-1^	θ_max_ = 28.0°, θ_min_ = 2.0°
ω and φ scans	*h* = −12→13
Absorption correction: multi-scan (*CrystalClear-SM Expert*; Rigaku, 2009)	*k* = −12→12
*T*_min_ = 0.958, *T*_max_ = 0.979	*l* = −24→24
18792 measured reflections	

### Refinement

**Table:**

Refinement on *F*^2^	Secondary atom site location: difference Fourier map
Least-squares matrix: full	Hydrogen site location: inferred from neighbouring sites
*R*[*F*^2^ > 2σ(*F*^2^)] = 0.041	H-atom parameters constrained
*wR*(*F*^2^) = 0.103	*w* = 1/[σ^2^(*F*_o_^2^) + (0.0361*P*)^2^] where *P* = (*F*_o_^2^ + 2*F*_c_^2^)/3
*S* = 0.85	(Δ/σ)_max_ < 0.001
4382 reflections	Δρ_max_ = 0.35 e Å^−^^3^
237 parameters	Δρ_min_ = −0.30 e Å^−^^3^
0 restraints	Extinction correction: *SHELXL97* (Sheldrick, 2008), Fc^*^=kFc[1+0.001xFc^2^λ^3^/sin(2θ)]^-1/4^
Primary atom site location: structure-invariant direct methods	Extinction coefficient: 0.0033 (5)

### Special details

**Table:**

Geometry. All e.s.d.'s (except the e.s.d. in the dihedral angle between two l.s. planes) are estimated using the full covariance matrix. The cell e.s.d.'s are taken into account individually in the estimation of e.s.d.'s in distances, angles and torsion angles; correlations between e.s.d.'s in cell parameters are only used when they are defined by crystal symmetry. An approximate (isotropic) treatment of cell e.s.d.'s is used for estimating e.s.d.'s involving l.s. planes.
Refinement. Refinement of *F*^2^ against ALL reflections. The weighted *R*-factor *wR* and goodness of fit *S* are based on *F*^2^, conventional *R*-factors *R* are based on *F*, with *F* set to zero for negative *F*^2^. The threshold expression of *F*^2^ > σ(*F*^2^) is used only for calculating *R*-factors(gt) *etc*. and is not relevant to the choice of reflections for refinement. *R*-factors based on *F*^2^ are statistically about twice as large as those based on *F*, and *R*- factors based on ALL data will be even larger.

### Fractional atomic coordinates and isotropic or equivalent isotropic displacement parameters (Å^2^)

**Table:**

	*x*	*y*	*z*	*U*_iso_*/*U*_eq_	
Cl1	−0.03716 (5)	1.21324 (5)	0.09402 (3)	0.03050 (16)	
N1	0.12552 (15)	0.71133 (16)	0.10904 (7)	0.0212 (4)	
N2	0.54914 (18)	0.70088 (18)	0.00901 (9)	0.0377 (5)	
C1	0.13022 (19)	0.84896 (19)	0.09184 (9)	0.0212 (4)	
C2	0.0363 (2)	0.94986 (19)	0.10034 (9)	0.0231 (5)	
H2	−0.0485	0.9312	0.1184	0.028*	
C3	0.0728 (2)	1.0788 (2)	0.08114 (9)	0.0238 (5)	
C4	0.19515 (19)	1.1085 (2)	0.05278 (9)	0.0260 (5)	
H4	0.2159	1.1992	0.0403	0.031*	
C5	0.2855 (2)	1.0062 (2)	0.04304 (10)	0.0251 (5)	
H5	0.3684	1.0253	0.0230	0.030*	
C6	0.25444 (19)	0.8741 (2)	0.06279 (9)	0.0216 (4)	
C7	0.32207 (19)	0.7456 (2)	0.06210 (9)	0.0230 (5)	
C8	0.24230 (19)	0.6490 (2)	0.09126 (9)	0.0236 (5)	
C9	0.27113 (19)	0.50407 (19)	0.10918 (10)	0.0252 (5)	
H9A	0.3431	0.4712	0.0795	0.030*	
H9B	0.1899	0.4493	0.0966	0.030*	
C10	0.31403 (19)	0.4831 (2)	0.18884 (10)	0.0235 (5)	
C11	0.4009 (2)	0.5740 (2)	0.22559 (11)	0.0303 (5)	
H11	0.4327	0.6509	0.2010	0.036*	
C12	0.4411 (2)	0.5531 (2)	0.29759 (11)	0.0371 (6)	
H12	0.5007	0.6154	0.3221	0.044*	
C13	0.3951 (2)	0.4425 (2)	0.33395 (11)	0.0387 (6)	
H13	0.4223	0.4290	0.3835	0.046*	
C14	0.3092 (2)	0.3513 (2)	0.29807 (11)	0.0397 (6)	
H14	0.2778	0.2746	0.3229	0.048*	
C15	0.2687 (2)	0.3716 (2)	0.22582 (11)	0.0344 (5)	
H15	0.2095	0.3087	0.2015	0.041*	
C16	0.4488 (2)	0.7192 (2)	0.03379 (10)	0.0268 (5)	
C17	0.00947 (19)	0.6386 (2)	0.12952 (9)	0.0220 (4)	
C18	−0.0077 (2)	0.6062 (2)	0.20068 (10)	0.0328 (5)	
H18	0.0541	0.6378	0.2381	0.039*	
C19	−0.1160 (2)	0.5272 (2)	0.21686 (11)	0.0384 (6)	
H19	−0.1279	0.5051	0.2659	0.046*	
C20	−0.2076 (2)	0.4795 (2)	0.16341 (11)	0.0290 (5)	
C21	−0.1911 (2)	0.5178 (2)	0.09294 (10)	0.0286 (5)	
H21	−0.2551	0.4898	0.0557	0.034*	
C22	−0.0833 (2)	0.59617 (19)	0.07563 (10)	0.0249 (5)	
H22	−0.0730	0.6208	0.0268	0.030*	
C23	−0.3186 (2)	0.3845 (2)	0.18130 (12)	0.0472 (7)	
H23A	−0.3989	0.4038	0.1496	0.071*	
H23B	−0.3389	0.3974	0.2318	0.071*	
H23C	−0.2904	0.2906	0.1741	0.071*	

### Atomic displacement parameters (Å^2^)

**Table:**

	*U*^11^	*U*^22^	*U*^33^	*U*^12^	*U*^13^	*U*^23^
Cl1	0.0339 (3)	0.0245 (3)	0.0329 (3)	0.0083 (3)	0.0016 (2)	0.0004 (2)
N1	0.0207 (9)	0.0196 (9)	0.0233 (8)	0.0016 (8)	0.0017 (7)	−0.0009 (7)
N2	0.0329 (11)	0.0306 (11)	0.0507 (12)	0.0012 (10)	0.0121 (9)	−0.0055 (9)
C1	0.0258 (11)	0.0186 (10)	0.0186 (9)	−0.0005 (10)	−0.0025 (8)	−0.0004 (8)
C2	0.0237 (11)	0.0250 (11)	0.0201 (10)	0.0017 (9)	−0.0013 (8)	−0.0017 (9)
C3	0.0273 (12)	0.0218 (11)	0.0218 (10)	0.0062 (10)	−0.0027 (8)	−0.0017 (8)
C4	0.0303 (12)	0.0212 (11)	0.0259 (10)	0.0000 (10)	−0.0011 (9)	0.0008 (9)
C5	0.0251 (12)	0.0255 (12)	0.0248 (10)	−0.0005 (10)	0.0014 (8)	−0.0002 (9)
C6	0.0227 (11)	0.0211 (11)	0.0205 (10)	0.0016 (9)	−0.0016 (8)	−0.0009 (8)
C7	0.0214 (11)	0.0232 (12)	0.0245 (10)	0.0023 (9)	0.0014 (8)	−0.0032 (8)
C8	0.0266 (12)	0.0221 (11)	0.0217 (9)	0.0052 (10)	−0.0012 (8)	−0.0053 (9)
C9	0.0249 (12)	0.0218 (11)	0.0287 (11)	0.0001 (10)	0.0004 (9)	−0.0045 (9)
C10	0.0198 (11)	0.0211 (11)	0.0297 (11)	0.0053 (9)	0.0020 (8)	−0.0014 (9)
C11	0.0317 (13)	0.0227 (12)	0.0360 (12)	0.0037 (10)	−0.0008 (10)	0.0023 (9)
C12	0.0389 (14)	0.0317 (13)	0.0387 (13)	0.0050 (11)	−0.0107 (10)	−0.0059 (11)
C13	0.0474 (15)	0.0377 (14)	0.0303 (12)	0.0129 (12)	−0.0036 (11)	0.0014 (10)
C14	0.0456 (15)	0.0351 (14)	0.0387 (13)	0.0022 (13)	0.0044 (11)	0.0105 (11)
C15	0.0350 (13)	0.0285 (13)	0.0394 (12)	−0.0016 (11)	0.0005 (10)	0.0007 (10)
C16	0.0317 (13)	0.0177 (11)	0.0310 (11)	0.0018 (10)	0.0011 (9)	−0.0025 (9)
C17	0.0230 (11)	0.0197 (11)	0.0234 (10)	0.0043 (9)	0.0023 (8)	0.0028 (8)
C18	0.0313 (13)	0.0446 (14)	0.0222 (11)	0.0006 (11)	−0.0016 (9)	−0.0002 (10)
C19	0.0360 (14)	0.0543 (16)	0.0259 (11)	0.0043 (13)	0.0097 (10)	0.0115 (11)
C20	0.0264 (12)	0.0227 (12)	0.0387 (12)	0.0083 (10)	0.0092 (10)	0.0043 (10)
C21	0.0289 (12)	0.0266 (12)	0.0300 (11)	0.0008 (10)	0.0004 (9)	−0.0045 (9)
C22	0.0304 (12)	0.0239 (11)	0.0207 (10)	0.0009 (10)	0.0032 (8)	0.0006 (9)
C23	0.0372 (15)	0.0389 (15)	0.0681 (17)	0.0018 (13)	0.0230 (12)	0.0092 (13)

### Geometric parameters (Å, °)

**Table:**

Cl1—C3	1.7487 (19)	C11—C12	1.383 (3)
N1—C8	1.379 (2)	C11—H11	0.9500
N1—C1	1.395 (2)	C12—C13	1.377 (3)
N1—C17	1.437 (2)	C12—H12	0.9500
N2—C16	1.146 (2)	C13—C14	1.382 (3)
C1—C2	1.385 (2)	C13—H13	0.9500
C1—C6	1.409 (2)	C14—C15	1.387 (3)
C2—C3	1.375 (2)	C14—H14	0.9500
C2—H2	0.9500	C15—H15	0.9500
C3—C4	1.396 (3)	C17—C18	1.379 (2)
C4—C5	1.374 (2)	C17—C22	1.381 (2)
C4—H4	0.9500	C18—C19	1.384 (3)
C5—C6	1.393 (3)	C18—H18	0.9500
C5—H5	0.9500	C19—C20	1.385 (3)
C6—C7	1.436 (3)	C19—H19	0.9500
C7—C8	1.376 (3)	C20—C21	1.381 (3)
C7—C16	1.428 (3)	C20—C23	1.506 (3)
C8—C9	1.490 (3)	C21—C22	1.382 (3)
C9—C10	1.523 (2)	C21—H21	0.9500
C9—H9A	0.9900	C22—H22	0.9500
C9—H9B	0.9900	C23—H23A	0.9800
C10—C15	1.387 (3)	C23—H23B	0.9800
C10—C11	1.393 (3)	C23—H23C	0.9800
			
C8—N1—C1	109.63 (16)	C10—C11—H11	119.8
C8—N1—C17	123.60 (16)	C13—C12—C11	120.4 (2)
C1—N1—C17	125.86 (16)	C13—C12—H12	119.8
C2—C1—N1	129.70 (18)	C11—C12—H12	119.8
C2—C1—C6	122.68 (18)	C12—C13—C14	119.8 (2)
N1—C1—C6	107.61 (17)	C12—C13—H13	120.1
C3—C2—C1	116.10 (18)	C14—C13—H13	120.1
C3—C2—H2	121.9	C13—C14—C15	120.1 (2)
C1—C2—H2	121.9	C13—C14—H14	119.9
C2—C3—C4	123.12 (19)	C15—C14—H14	119.9
C2—C3—Cl1	118.98 (16)	C14—C15—C10	120.5 (2)
C4—C3—Cl1	117.89 (15)	C14—C15—H15	119.7
C5—C4—C3	119.79 (19)	C10—C15—H15	119.7
C5—C4—H4	120.1	N2—C16—C7	177.6 (2)
C3—C4—H4	120.1	C18—C17—C22	120.01 (19)
C4—C5—C6	119.39 (19)	C18—C17—N1	121.59 (17)
C4—C5—H5	120.3	C22—C17—N1	118.34 (16)
C6—C5—H5	120.3	C17—C18—C19	119.23 (19)
C5—C6—C1	118.87 (18)	C17—C18—H18	120.4
C5—C6—C7	135.07 (18)	C19—C18—H18	120.4
C1—C6—C7	106.05 (17)	C18—C19—C20	121.69 (19)
C8—C7—C16	124.79 (18)	C18—C19—H19	119.2
C8—C7—C6	108.70 (16)	C20—C19—H19	119.2
C16—C7—C6	126.47 (18)	C21—C20—C19	117.9 (2)
C7—C8—N1	107.99 (17)	C21—C20—C23	121.1 (2)
C7—C8—C9	129.96 (18)	C19—C20—C23	120.98 (19)
N1—C8—C9	121.84 (18)	C20—C21—C22	121.22 (19)
C8—C9—C10	112.56 (15)	C20—C21—H21	119.4
C8—C9—H9A	109.1	C22—C21—H21	119.4
C10—C9—H9A	109.1	C17—C22—C21	119.84 (18)
C8—C9—H9B	109.1	C17—C22—H22	120.1
C10—C9—H9B	109.1	C21—C22—H22	120.1
H9A—C9—H9B	107.8	C20—C23—H23A	109.5
C15—C10—C11	118.79 (19)	C20—C23—H23B	109.5
C15—C10—C9	120.45 (18)	H23A—C23—H23B	109.5
C11—C10—C9	120.75 (18)	C20—C23—H23C	109.5
C12—C11—C10	120.42 (19)	H23A—C23—H23C	109.5
C12—C11—H11	119.8	H23B—C23—H23C	109.5
			
C8—N1—C1—C2	−179.86 (18)	C17—N1—C8—C9	−15.6 (3)
C17—N1—C1—C2	10.8 (3)	C7—C8—C9—C10	97.8 (2)
C8—N1—C1—C6	−0.43 (19)	N1—C8—C9—C10	−76.2 (2)
C17—N1—C1—C6	−169.82 (15)	C8—C9—C10—C15	140.01 (19)
N1—C1—C2—C3	177.05 (17)	C8—C9—C10—C11	−40.9 (2)
C6—C1—C2—C3	−2.3 (3)	C15—C10—C11—C12	0.1 (3)
C1—C2—C3—C4	1.7 (3)	C9—C10—C11—C12	−178.96 (17)
C1—C2—C3—Cl1	−177.16 (13)	C10—C11—C12—C13	−0.4 (3)
C2—C3—C4—C5	−0.1 (3)	C11—C12—C13—C14	0.6 (3)
Cl1—C3—C4—C5	178.81 (14)	C12—C13—C14—C15	−0.4 (3)
C3—C4—C5—C6	−1.0 (3)	C13—C14—C15—C10	0.1 (3)
C4—C5—C6—C1	0.5 (3)	C11—C10—C15—C14	0.0 (3)
C4—C5—C6—C7	−178.58 (19)	C9—C10—C15—C14	179.10 (18)
C2—C1—C6—C5	1.3 (3)	C8—N1—C17—C18	89.9 (2)
N1—C1—C6—C5	−178.18 (15)	C1—N1—C17—C18	−102.1 (2)
C2—C1—C6—C7	−179.42 (16)	C8—N1—C17—C22	−87.5 (2)
N1—C1—C6—C7	1.11 (19)	C1—N1—C17—C22	80.5 (2)
C5—C6—C7—C8	177.7 (2)	C22—C17—C18—C19	2.2 (3)
C1—C6—C7—C8	−1.4 (2)	N1—C17—C18—C19	−175.10 (18)
C5—C6—C7—C16	−4.4 (3)	C17—C18—C19—C20	0.1 (3)
C1—C6—C7—C16	176.51 (17)	C18—C19—C20—C21	−2.6 (3)
C16—C7—C8—N1	−176.80 (16)	C18—C19—C20—C23	175.09 (19)
C6—C7—C8—N1	1.2 (2)	C19—C20—C21—C22	2.9 (3)
C16—C7—C8—C9	8.5 (3)	C23—C20—C21—C22	−174.81 (18)
C6—C7—C8—C9	−173.51 (17)	C18—C17—C22—C21	−1.9 (3)
C1—N1—C8—C7	−0.5 (2)	N1—C17—C22—C21	175.46 (17)
C17—N1—C8—C7	169.21 (15)	C20—C21—C22—C17	−0.7 (3)
C1—N1—C8—C9	174.73 (16)		

### Hydrogen-bond geometry (Å, °)

**Table:**

Cg is the centroid of the C17–C22 ring.

**Table:**

*D*—H···*A*	*D*—H	H···*A*	*D*···*A*	*D*—H···*A*
C9—H9A···N2^i^	0.99	2.64	3.557 (3)	154
C14—H14···Cg^ii^	0.95	2.84	3.735 (2)	157

Symmetry codes: (i) −*x*+1, −*y*+1, −*z*; (ii) −*x*, *y*−1/2, −*z*+1/2.
